# ELYFANT Study Protocol: An Observational Study to Assess Awareness of and Adherence to Advice on Early Introduction of Food Allergens and Whether It Is Preventing Food Allergy

**DOI:** 10.1111/cea.70277

**Published:** 2026-03-12

**Authors:** Raquel Granell, Mary Feeney, Thisanayagam Umasunthar, Rachel L. Peters, Jennifer Koplin, Matthew J. Ridd

**Affiliations:** ^1^ Centre for Applied Excellence in Skin & Allergy Research (CAESAR), Centre for Academic Primary Care, NIHR School for Primary Care Research University of Bristol Bristol UK; ^2^ Murdoch Children's Research Institute Parkville Victoria Australia; ^3^ Department of Paediatrics the University of Melbourne Parkville Victoria Australia; ^4^ University of Queensland St Lucia Queensland Australia

## Abstract

- ELYFANT is the first UK study of awareness, implementation and outcomes of early allergen introduction advice.
- Strategies for implementation of existing evidence will be evaluated and findings will inform if any modifications are needed.

To the Editor,

Food allergy (FA) is an immune‐mediated hypersensitivity reaction to food that affects ~5% of children in the United Kingdom [1]. Among the most common food allergies are cow's milk, egg, wheat, sesame, peanut, tree nuts and fish. There is good evidence for some allergens that early introduction into the infant diet reduces the risk of food allergy [2].

UK guidelines that recommended deliberate delayed introduction of allergenic foods were withdrawn in 2008; since then, there has been a shift towards recommending active introduction in the first year of life [3].

In 2018, following a review of scientific evidence, a joint committee of the UK's Scientific Advisory Committee on Nutrition (SACN) and the Committee on Toxicity of Chemicals in Food, Consumer Products and the Environment (COT) published a statement recommending the introduction of complementary foods containing peanut and hen's egg from around 6 months of age [4]. It further specified that the deliberate exclusion of peanut or hen's egg beyond 6–12 months of age may increase the risk of allergy to the same foods. The British Society of Allergy and Clinical Immunology (BSACI) published guidance in the same year, also recommending egg and peanut introduction from around 6 months for most infants, adding that infants at high risk of food allergy may benefit from earlier introduction from 4 months of age alongside other complementary foods [5].

There are no published contemporaneous studies of awareness or uptake of the change in guidance in the UK and whether it is having an effect on the prevalence of food allergy. In the EarLY Food Allergens iNTroduction (ELYFANT) study (Table 1), we assess current understanding, implementation and outcomes of early allergen introduction advice in England. The full protocol is available from the Bristol Research Portal [6].

**TABLE 1:** Summary of topics collected in ELYFANT 2025–2026 survey.

Questionnaire	1	2
Babies' age IQR (months)	15–16	22–23
Start‐end date	March–May 2025	October 2025–January 2026
Estimated number at completion	~2000	~1600
TOPICS		
Mothers' main language	●	●
Child's sex at birth	●	●
Child's age at completion	●	●
Family history allergy	●	●
Breastfeeding and reasons for stopping	●	●
Common allergens in current diet	●	●
Introduction of common allergens (first time, continuity/intermittence)	●	
Currently avoiding common allergens and why	●	●
Child suspected/confirmed food allergy	●	●
Prevention of food allergy	●	
Awareness of government advice (peanut and hen's egg)	●	
Medical history (development of eczema, asthma)	●	●
Common infant symptoms	●	●

We recruited mothers through the 2024 Infant Feeding Survey (IFS) [7]. The IFS is commissioned by the Department of Health and Social Care and the 2023 study (the ninth national survey of infant feeding practices) and delivered by Ipsos Limited. It is based on a representative sample of mothers from all births in England in late 2023. The aim of the IFS is to provide information on infant feeding behaviours including prevalence and duration of breastfeeding, the use of foods and drinks other than breastmilk in infancy, as well as data on some broader lifestyle behaviours. More than 20,000 mothers were contacted by IFS, and data have been collected at three different timepoints: at 9–12 weeks, 4–6 months and 8–10 months.

Mothers who took part in the last IFS survey (8–10 months) were asked for permission to send them two further questionnaires: when their child was 15–17 months old, and when their child was 22–24 months old. Each questionnaire asks about the child's diet and whether they have developed food allergy or any related conditions, such as eczema or asthma. The second questionnaire also asked mothers if they were willing to take part in an optional interview where we plan to gain a more in‐depth and complete understanding of knowledge about early introduction advice and outcomes. This will inform antenatal/postnatal information given to parents, training for HCPs and/or future research to support early introduction.

Table 1 summarises the topics collected in ELYFANT. Data analysis will include description of the population demographics with a focus on sources of advice, awareness of change of government advice about when to introduce peanut and hen's egg into their child's diet, timing of introduction of common allergens, frequency and continuity/intermittence of ingestion, and reasons for avoiding common food allergens.

We will identify predictors of early introduction of common allergens and assess associations between timing of introduction and reported allergy diagnosis using logistic regression methods after adjusting for possible confounders identified through a directed acyclic graph. Prevalence of reported food allergy diagnosis and co‐morbidities (eczema, wheeze/asthma) in this sample will be estimated and compared with other similar cohorts. We will explore patterns of allergen ingestion (continuity vs. intermittence) using Sankey diagrams.

Mothers who complete the questionnaires and those that were lost in the follow up will be compared for demographic differences, and we will aim to assess the validity of our research findings by exploring potential recall bias using the longitudinal data structure. Validity of parent‐reported diagnosis by a doctor or nurse or health professional will be examined in a sensitivity analysis including parent report of suspected food allergy.

We will interview a sub‐sample of survey respondents to gain a more in‐depth and complete understanding of knowledge about early introduction advice and outcomes. Data will be analysed thematically. This will inform antenatal/postnatal information given to parents, training for professionals and/or future research to support early introduction.

Based on an estimated sample size of 1600–2000 mothers, we might be able to detect an absolute difference of 0.8%–0.9%, that is a decline in food allergy prevalence from a baseline prevalence of 2% [8] to 1.1%–1.2% at 80% power.

Ethical approval has been obtained from the University of Bristol Research Ethics (Ref 23390). Data analysis, interpretation and conclusions will be presented at national and international conferences, published in peer‐reviewed journals and disseminated via social media.

This is the first study in the UK to assess current infant feeding practices with regard to allergy prevention. Our findings will inform whether the community is following the recommended advice or if further strategies are needed to improve uptake.

## Author Contributions

Matthew J. Ridd and Raquel Granell conceived and designed the study. Matthew J. Ridd obtained the funding. All authors contributed to the development of the protocol. Raquel Granell drafted the research letter. All authors have read and approved the final manuscript and have agreed to publication.

## Funding

This study is funded by an NIHR Professorship (NIHR303123). The views expressed are those of the author(s) and not necessarily those of the NIHR or the Department of Health and Social Care.

## Conflicts of Interest

The authors declare no conflicts of interest.

## Data Availability

Anonymised data from ELYFANT will be stored securely for future analysis on a research data facility. Requests for access to data must be via a written confidentiality and data‐sharing agreement, which will cover limitations of use, transfer to third parties and storage. Anyone applying for use of the data will be scrutinised for appropriate eligibility. ELYFANT objectives and data collected in ELYFANT are listed in a table included in the full protocol. This table and all study documents including the ELYFANT questionnaire at 15 months are available from the full protocol in the Bristol Research Portal.
